# P-1760. Analysis of the gut microbiome in pediatric allogeneic hematopoietic stem cell transplantation: pilot study

**DOI:** 10.1093/ofid/ofaf695.1931

**Published:** 2026-01-11

**Authors:** Euri Seo, Eun Seok Choi, Jung Hwa Kim, Hyery Kim, Kyung-Nam Koh, Ho Joon Im, Jina Lee

**Affiliations:** Department of Pediatrics, Asan Medical Center Children’s Hospital, University of Ulsan College of Medicine, Seoul, Seoul-t'ukpyolsi, Republic of Korea; Department of Pediatrics, Asan Medical Center Children’s Hospital, University of Ulsan College of Medicine, Seoul, Seoul-t'ukpyolsi, Republic of Korea; Department of Pediatrics, Asan Medical Center Children’s Hospital, University of Ulsan College of Medicine, Seoul, Seoul-t'ukpyolsi, Republic of Korea; Department of Pediatrics, Asan Medical Center Children’s Hospital, University of Ulsan College of Medicine, Seoul, Seoul-t'ukpyolsi, Republic of Korea; Department of Pediatrics, Asan Medical Center Children’s Hospital, University of Ulsan College of Medicine, Seoul, Seoul-t'ukpyolsi, Republic of Korea; Department of Pediatrics, Asan Medical Center Children’s Hospital, University of Ulsan College of Medicine, Seoul, Seoul-t'ukpyolsi, Republic of Korea; Asan medical center, Seoul, Seoul-t'ukpyolsi, Republic of Korea

## Abstract

**Background:**

The changes in the gut microbiome of pediatric hematopoietic stem cell transplantation (HSCT) recipients and its association with the development of acute graft-versus host disease (aGvHD) have not been well investigated. We conducted a prospective pilot study with a small group of pediatric HSCT recipients to evaluate changes in the gut microbiome of recipients before and after HSCT, assess the associations with the development of aGvHD, and explore the potential link between the corresponding donors’ microbiome and the development of aGvHD in HSCT recipients.

**Figure:**
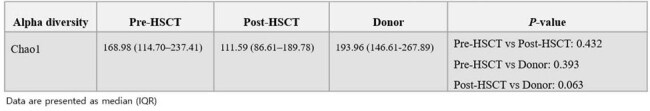
Chao1 index of the gut microbiome in recipients pre-HSCT and post-HSCT and donors

**Figure:**
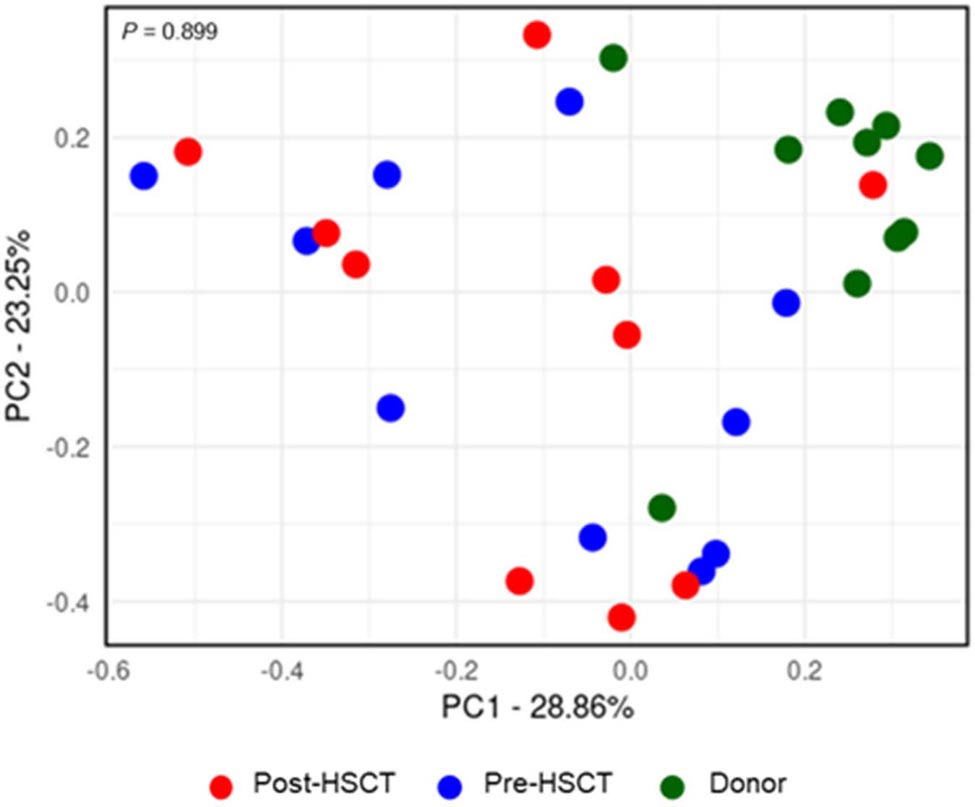
Principal coordinates analysis based on Bray–Curtis distances of stool microbiome of the recipients and donors

**Methods:**

We collected one fecal sample from the HSCT recipient and one from their relevant donor before transplantation, and another from the recipient one month after HSCT. We assessed alpha diversity, beta diversity, and relative abundance obtained through 16S rDNA sequencing in each sample and compared these measures between recipients with and without aGvHD.

**Figure:**
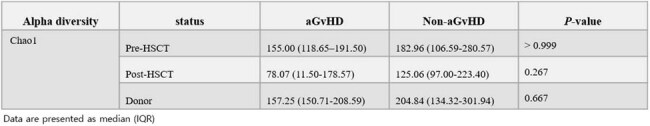
Alpha diversity indices of the gut microbiome in recipients pre-HSCT and post-HSCT and donors based on aGvHD status

**Figure:**
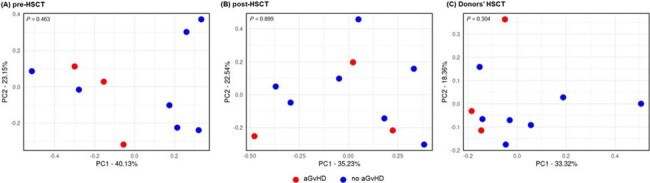
Beta diversity by principal coordinates analysis derived from Bray–Curtis distances according to the presence or absence of aGvHD. (A) pre-HSCT. (B) post-HSCT. (C) donor.

**Results:**

Among the 10 HSCT recipients, 2 developed skin aGvHD of grade 2 and grade 1, respectively, and 1 developed grade 3 gastrointestinal and skin aGvHD. The alpha diversity of the post-HSCT gut microbiome was reduced compared to that of the pre-HSCT gut microbiome and was also lower than that of the donor. The alpha diversity of the pre-HSCT and post-HSCT gut microbiome in recipients with aGvHD, as well as those of their donors, tended to be lower than in recipients without aGvHD. There were no significant differences in the beta diversity of the pre-HSCT and post-HSCT recipients' microbiomes and the donors' microbiomes according to aGvHD. In the aGvHD group, the post-HSCT gut microbiome showed a lower abundance of the *Erysipelatoclostridium* genus compared to the non-aGvHD group. Additionally, the pre-HSCT gut microbiome in the aGvHD group had a greater abundance of *Raoultella ornithinolytica* B6 species. Donors whose recipients developed aGvHD had a higher abundance of the *Eubacterium eligens* group and the *Veillonellaceae* family.

**Conclusion:**

Evaluating the gut microbiome of recipients before and after transplantation, as well as that of their donors, may help predict the occurrence of aGvHD in recipients.

**Disclosures:**

All Authors: No reported disclosures

